# Chemotherapy Sensitizes Colon Cancer Initiating Cells to Vγ9Vδ2 T Cell-Mediated Cytotoxicity

**DOI:** 10.1371/journal.pone.0065145

**Published:** 2013-06-06

**Authors:** Matilde Todaro, Valentina Orlando, Giuseppe Cicero, Nadia Caccamo, Serena Meraviglia, Giorgio Stassi, Francesco Dieli

**Affiliations:** 1 Dipartimento di Discipline Chirurgiche ed Anatomiche, Università di Palermo, Palermo, Italy; 2 Biomedical Research Centre, Università di Palermo, Palermo, Italy; 3 Dipartimento di Biopatologia e Biotecnologie Mediche e Forensi, Università di Palermo, Palermo, Italy; Centre de Recherche Public de la Santé (CRP-Santé), Luxembourg

## Abstract

Colon cancer comprises a small population of cancer initiating stem cells (CIC) that is responsible for tumor maintenance and resistance to anti-cancer therapies, possibly allowing for tumor recapitulation once treatment stops. Combinations of immune-based therapies with chemotherapy and other anti-tumor agents may be of significant clinical benefit in the treatment of colon cancer. However, cellular immune-based therapies have not been experimented yet in the population of colon CICs. Here, we demonstrate that treatment with low concentrations of commonly used chemotherapeutic agents, 5-fluorouracyl and doxorubicin, sensitize colon CICs to Vγ9Vδ2 T cell cytotoxicity. Vγ9Vδ2 T cell cytotoxicity was largely mediated by TRAIL interaction with DR5, following NKG2D-dependent recognition of colon CIC targets. We conclude that *in vivo* activation of Vγ9Vδ2 T cells or adoptive administration of *ex-vivo* expanded Vγ9Vδ2 T cells at suitable intervals after chemotherapy may substantially increase anti-tumor activities and represent a novel strategy for colon cancer immunotherapy.

## Introduction

In recent years, novel insights in cancer research have suggested that the capacity to initiate and sustain tumor growth is a unique characteristic of a small subset of cancer cells with stemness properties within the tumor mass, called “cancer stem cells” (CSCs) or “cancer-initiating cells” (CICs) [1]. Chemotherapy remains the primary treatment choice for many advanced cancers and has cytotoxic anti-tumor activity through a range of mechanisms. However, most cancers are resistant to current therapies due to the slow-cycling CICs, the location of these cells within hypoxic niches [2], [3], and because the malignant cells have the capacity to develop mechanisms to resist or escape the cytotoxic effects of chemotherapy [4], which include up-regulation of several ATP-binding cassette transporters, active DNA-repair capacity and over-expression of anti-apoptotic molecules that cause changes in the signalling pathways controlling proliferation, differentiation and apoptosis [5].

Several studies have demonstrated that treatment of tumor cells with chemotherapeutic drugs induces or increases their sensitivity to cytotoxicity by NK or T lymphocytes; thus, combinations of cellular immune-based therapies with chemotherapy and other anti-tumor agents may be of significant clinical benefit in the treatment of many forms of cancer [6].

γδ T cells are of particular interest for use in such combined therapies due to their potent anti-tumor cytotoxicity and the relative ease of generation *in vitro* [7]. Human γδ T cells can be divided into two main populations based upon δ chain expression [8]: γδ T cells expressing the Vδ1 chain are most often found in mucosal tissues, where they are involved in maintaining epithelial tissue integrity in the face of damage, infection, or tumor transformation, while γδ T cells expressing the Vδ2 chain paired to the Vγ9 chain (here and thereafter called Vγ9Vδ2 T cells) predominate in the peripheral blood and secondary lymphoid organs [9]. While the ligand(s) recognized by Vδ1 cells remain unknown, Vγ9Vδ2 T cells recognize non peptidic antigens by a MHC-unrestricted mechanism, an important feature which distinguishes them from αβ T cells [9]. Specifically, Vγ9Vδ2 T cells recognize phosphoantigens that are produced through the isoprenoid biosynthesis pathways [10]–[12]. Phosphoantigens are not stimulatory at physiologic levels, but transformed and infected cells, produce increased levels of metabolic intermediates that are able to activate Vγ9Vδ2 T cells [13]–[15]. Accordingly, Vγ9Vδ2 T cells can also be activated, through an indirect mechanism, by aminobisphosphonates, a class of drugs used to treat certain bone diseases, that inhibit farnesyl pyrophosphate synthase, and cause accumulation of endogenous upstream metabolites such as isopentenylpyrophosphate (IPP) [16]. Vγ9Vδ2 T cells may indirectly contribute to the immune defense against cancer cells, by producing cytokines typical of Th1, Th2 or Th17 cells [17]–[19], or cross-talking with dendritic cells [20], macrophages [21] and B cells [22]–[24]. Additionally, Vγ9Vδ2 T cells perform direct potent cytotoxic activity toward cancer cells, which is mediated in much the same manner as for CD8 T cells and NK cells, through perforin/granzyme, Fas/FasL, TNF/TNF-R and TRAIL-TRAIL-R pathways [10].

In this study, we have assessed the potential synergy of combining chemotherapy and Vγ9Vδ2 T cell-mediated cytotoxicity for anti-tumor therapy. Specifically, as colon CICs are resistant to both chemotherapeutic drugs and to Vγ9Vδ2 T cell-mediated cytotoxicity, we have determined whether chemotherapy can be used to sensitize colon CIC targets to Vγ9Vδ2 T cell cytotoxicity, based on three lines of evidence: (1) pioneering work by Mattarollo and colleagues [25] has demonstrated high levels of cytotoxicity against solid tumor-derived cell lines with combination treatment utilizing Vγ9Vδ2 T cells and chemotherapeutic agents; (2) IL-17-producing γδ T cells play a decisive role in chemotherapy-induced anti-cancer immune responses in the mouse [26]; (3) treatment of colon CICs with the bisphosphonate zoledronate enhances their sensitivity to Vγ9Vδ2 T cell killing [27].

We show here that chemotherapeutic drugs currently used for treatment of colon cancer patients, 5-fluorouracyl and doxorubicin, are capable to sensitize colon CICs to Vγ9Vδ2 T cell-mediated killing and we demonstrate that the underlying mechanisms involve NKG2D and TRAIL.

## Results

### Resistance of Colon CICs to Chemotherapy

We have previously reported that colon cancer comprises a vast majority of differentiated cells and a small population of CICs that are responsible for tumor initiation and maintenance [28]. For this study purposes, we purified and propagated colon cancer spheres from surgical fragments of 5 patients with colon carcinoma. These cancer sphere lines were identified through the expression of CD133 and the epithelial specific antigen ESA, displayed adherence to the culture dishes in the presence of serum and subsequently differentiated into large, polygonal colon cells expressing colon epithelial markers, such as villin, suggesting that colon cancer spheres maintained the ability to *in vitro* differentiate in enterocyte-like cells. Most importantly, when injected subcutaneously into NOD/SCID mice, a low number of colon cancer spheres, but not sphere-derived differentiated cells, retained the capacity to form a tumor that closely resembled the human original tumor (**Supporting Figure S1**).

**Figure 1:**
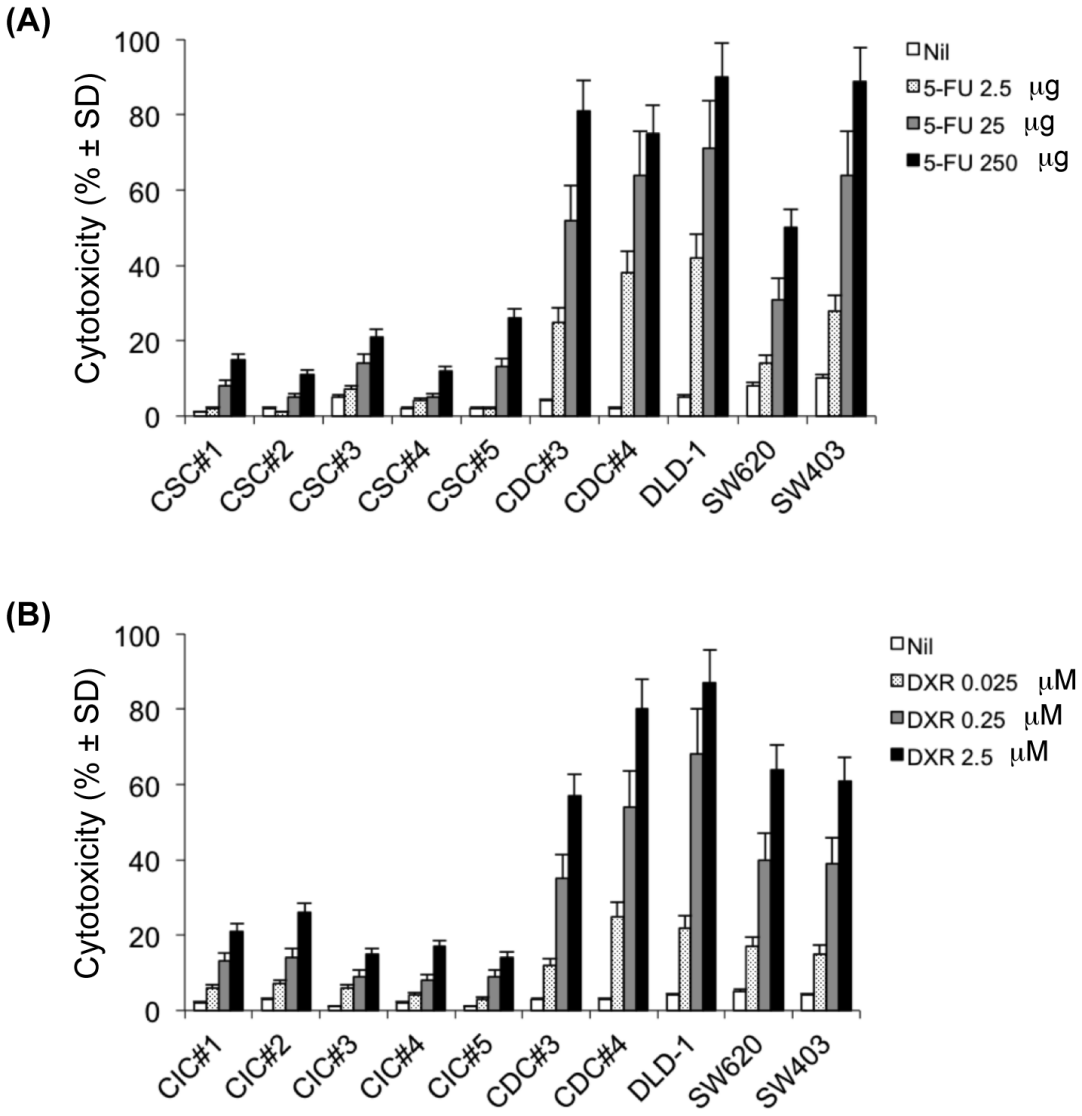
Cell cytotoxicity following treatment with 5-FU (A) or DXR (B). Colon CICs, differentiated colon cancer cell lines DLD-1, SW620, SW403, CDC#3 and CDC#4 were treated with different concentrations of 5-FU or DXR for 48 hrs. Cytotoxicity (% ± SD) was determined by the degree of reduction of viable cells with the ability to retain CFSE and exclude PI (CFSE^high^ PI^−^). Shown is a representative experiment out of three.

CICs are characterized by high resistance to drugs and general toxins which target rapidly proliferating cells and spare the slow dividing cells, due to an up-regulation of several ATP-binding cassette transporters, active DNA-repair capacity, over-expression of anti-apoptotic molecules that cause changes in the signalling pathways controlling proliferation, differentiation and apoptosis [5]. Accordingly, exposure of 5 different colon CIC lines (CIC#1 to CIC#5) to 5-FU (2.5 and 25 µg/ml) (**Figure 1A**) or DXR (0.025 and 0.25 µM) (**Figure 1B**) for 24–72 hrs had virtually no significant cytotoxic effect, as determined by PI staining. Highest doses of 5-FU (250 µg/ml) and DXR (2.5 µM) caused low, yet detectable cytotoxicity of CIC lines ranging from 15±5% to 23±6% (mean ± SD). Conversely, 5-FU and DXR were fully capable of killing 3 differentiated colon cancer cell lines DLD-1, SW620 and SW403, and 2 differentiated cell lines (CDC#3 and CDC#4) obtained from two patients (P#3 and P#4) where form the CICs lines were also obtained, with a dose-dependent increase in cytotoxicity up to 85%. The viability of untreated cells was all over 90% (**Figures 1A and B**).

### Chemotherapy Sensitizes Colon CICs to Vγ9Vδ2 T Cell Cytotoxicity

**Figure 2:**
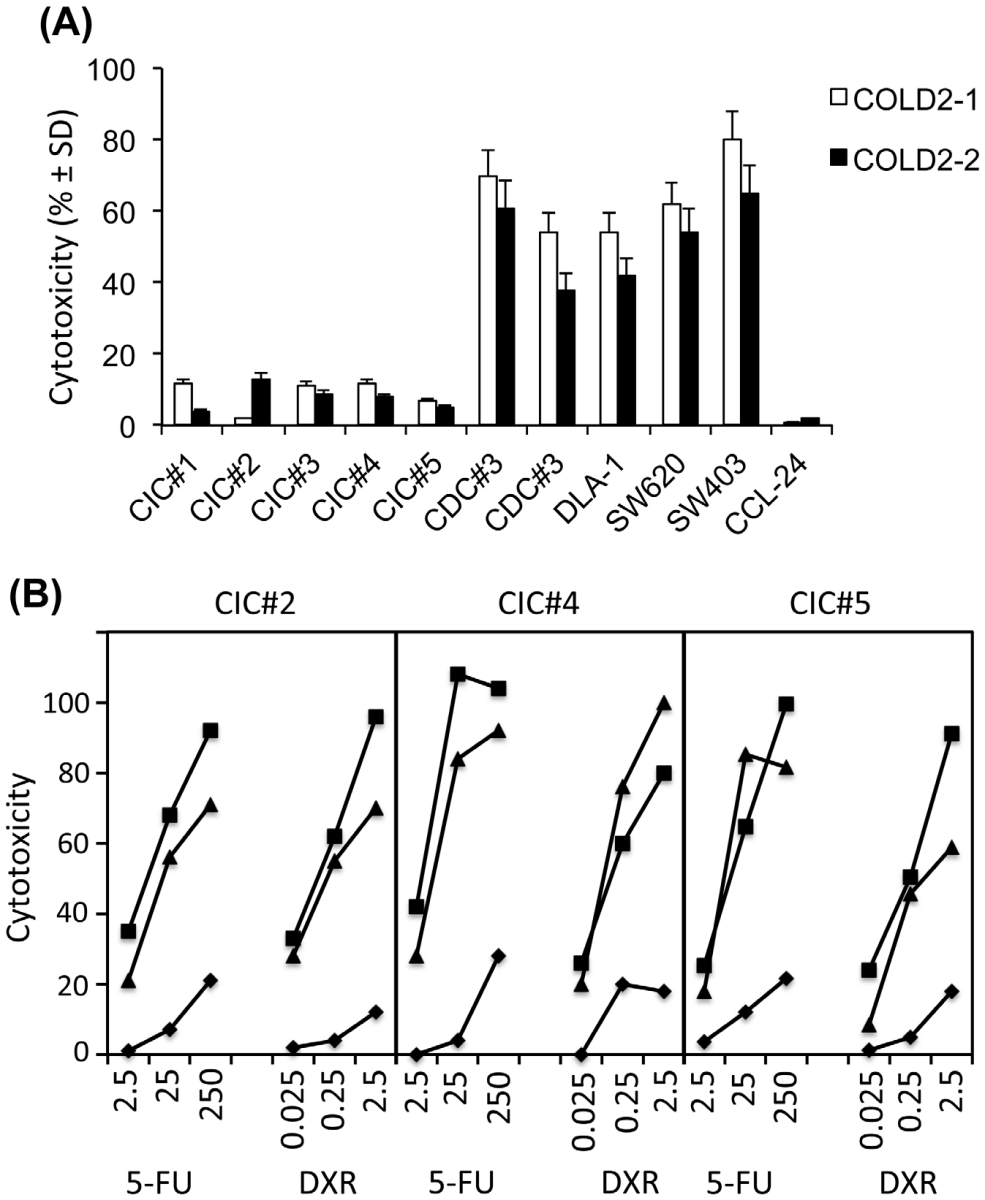
Chemotherapy sensitizes resistant colon CICs to Vγ9Vδ2 cell-mediated cytotoxicity. (A) Cytotoxicity percentage of 2 different to Vγ9Vδ2 T cell lines, COLD2-1 and COLD2-2 obtained from 2 patients affected by colon cancer, against colon cancer sphere cells from 5 different patients (CIC#1 to CIC#5), differentiated colon cancer cell lines DLD-1, SW620, SW403, CDC#3 and CDC#4, and the normal colon cell line CCL-241, at an E:T ratio of 50∶1. (B) Three different target colon CICs (CIC#2, CIC#4 and CIC#5) treated with or without either 5-FU (2.5 to 250 µg/ml) or DXR (0.025 to 2.5 µM) for 48 hrs were tested for their sensitivity to 2 different to Vγ9Vδ2 T cell lines, COLD2-1 and COLD2-2 obtained from 2 patients affected by colon cancer and used at an E:T ratio of 20∶1. Results indicate cytotoxicity of tumor targets following 6 hrs co-culture with Vγ9Vδ2 T cell lines. Data are mean percentage ± SD of 5 different experiments, each carried out in triplicate.

In analogy to their resistance to chemotherapy, the five tested colon CIC lines, were also resistant to Vγ9Vδ2 T cell-mediated cytotoxicity, even when an E:T ratio of 50∶1 was used (**Figure 2A**). The poor cytotoxic activity against colon CICs was not an intrinsic feature of the Vγ9Vδ2 T cells, because the differentiated colon cancer cell lines DLD-1, SW620, SW403, CDC#3 and CDC#4 were efficiently killed by two Vγ9Vδ2 T cell lines COLD2-1 and COLD2-2 obtained from two different colon cancer patients (P#3 and P#4) (**Figure 2A**), as well as Vγ9Vδ2 T cell lines obtained from healthy subjects (data not shown). As a control, all the tested Vγ9Vδ2 T cell lines failed to kill the normal colon cell line CCL-241 (**Figure 2A**).

In previous studies, we have demonstrated that zoledronate sensitizes colon cancer CICs to Vγ9Vδ2 T cell cytotoxicity [27]. The capability of Vγ9Vδ2 T cells to kill colon cancer CICs was then assessed after treatment of the targets with chemotherapy. Representative results obtained with three different CIC lines (CIC#2, CIC#4 and CIC#5) are shown in **Figure 2B**. Vγ9Vδ2 T cell cytotoxicity was enhanced in all cases by pre-treatment of target CICs with chemotherapy. In detail, almost complete lysis of CIC lines resulted from the combination of the highest doses of 5-FU (250 µg/ml) or DXR (2.5 µM) and Vγ9Vδ2 T cells, with cell death percentages over 90% at an E:T ratio of 20∶1. Treatment of targets with lower doses chemotherapy (2.5 and 25 µg/ml 5-FU and 0.025 and 0.25 µM DXR) resulted in enhanced killing of CIC lines by Vγ9Vδ2 T cells, indicating that chemotherapy and Vγ9Vδ2 T cells have additive activity even when used at suboptimal doses.

### Chemotherapy Upregulates DR5 (TRAIL-R2) Death Receptor Expression on CICs

**Figure 3:**
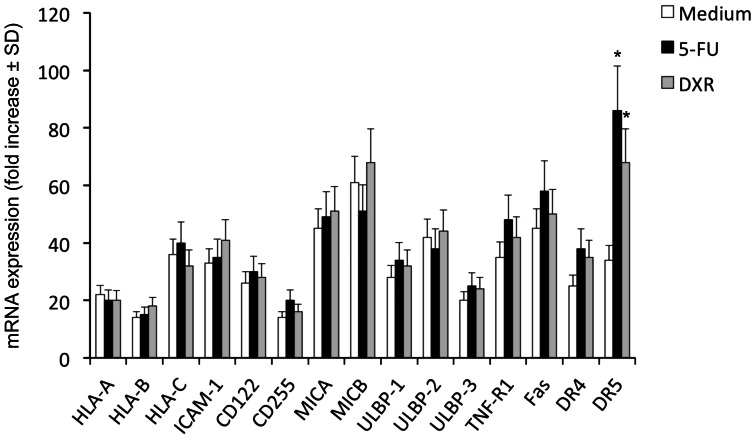
Colon CICs constitutively express molecules involved in by Vγ9Vδ2 T cell-mediated cytotoxicity: effect of chemotherapy. RT-PCR of the expression of mRNA encoding for different surface molecules in colon CICs treated with or without either 5-FU (25 µg/ml) or DXR (0.25 µM) for 48 hrs. Data represent the mean values ± SD of 4 separate experiments, each performed with colon cancer spheres from 5 different patients (CIC#1 to CIC#5).

To decipher the molecular mechanisms behind chemotherapy-mediated sensitization of CICs to Vγ9Vδ2 T cells cytotoxicity, we focused on expression of mRNA encoding for molecules known to be ligands for key activating receptors on Vγ9Vδ2 T cells and death receptors, before and after exposure of CICs to chemotherapy agents. As shown in **Figure 3**, all of these molecules were constitutively expressed in CICs, although expression consistently varied amongst different CIC lines; however, no major differences were observed in all tested CIC lines for HLA-class I, ICAM-1, CD155, CD112, MICA/B and ULPBP1–4 expression before and after exposure to chemotherapy agents.

**Figure 4:**
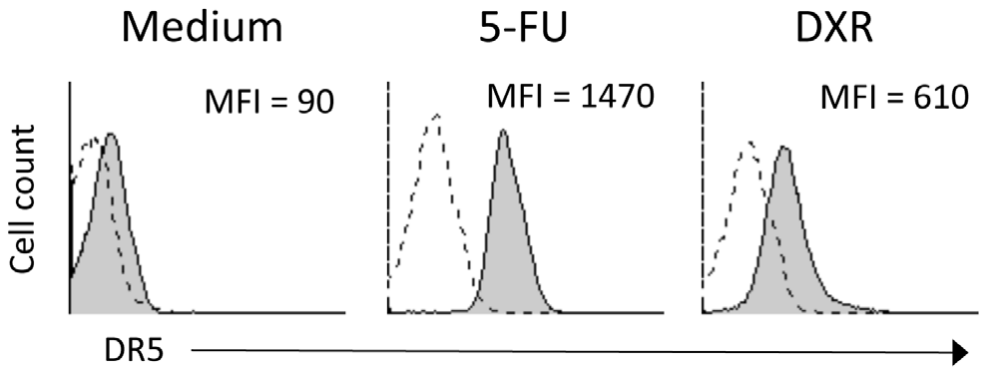
Chemotherapy upregulates DR5 expression on coloc CICs. Colon CICs were treated with medium, 5-FU (25 µg/ml) or DXR (0.25 µM) for 48 hrs, washed extensively and stained with anti-DR5 mAb. Flow cytometry histograms show DR5. Mean fluorescence intensity (MFI) for DR5 staining is indicated in the upper right corner of each panel. Dotted lines represent isotype control mAb, while grey filled histogram represent anti-DR5 mAb.

Expression of Fas (CD95), TNF-R1, DR4 (TRAIL-R1) and DR5 (TRAIL-R2) death receptors was increased in the majority of CIC lines following exposure to chemotherapeutic agents (**Figure 3**), but increased expression of Fas, TNF-R1 and DR4 did not attain statistical significance. The greatest and significant increase was only observed for DR5 expression after exposure of CICs to 5-FU and, although at a lesser extent, DXR (**Figure 3**). Upregulation of DR5 following 48 hrs exposure of colon CICs to chemotherapy was confirmed by flow cytometry upon staining with specific mAb (**Figure 4**).

### Killing of Chemotherapy-treated CICs by Vγ9Vδ2 T Cells is Mediated by NKG2D and TRAIL

**Figure 5:**
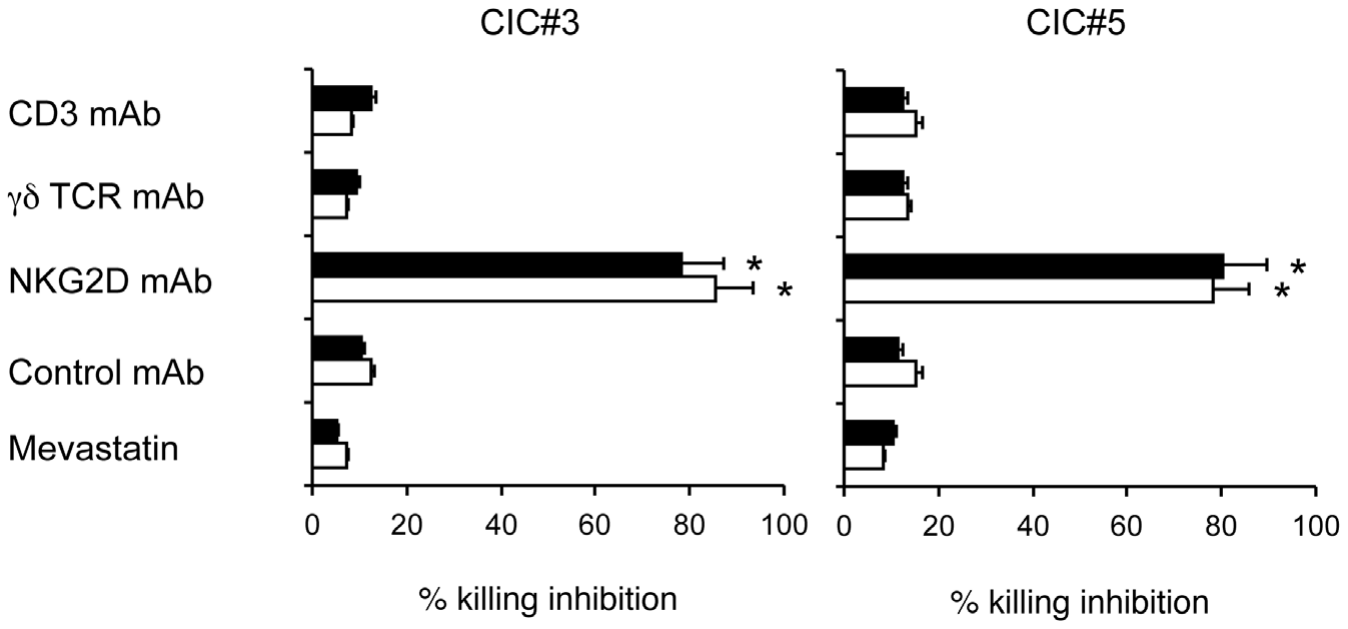
Modulation of the cytotoxic activity of Vγ9Vδ2 T cells by blocking the TCR or NKG2D interactions. The Vγ9Vδ2 T cell line COLD2-1 was cultured with two chemotherapy-treated colon CICs (CIC#3 and CIC#5) at an E:T ratio of 20∶1, in the presence of blocking antibodies to the γδ TCR, CD3, NKG2D, or in the presence of mevastatin. Specific cytotoxicity levels achieved by the Vγ9Vδ2 T cell line COLD2-1 were 65±11 for CIC#3 and 71±9 for CIC#5. Data are mean ± SD of two experiments carried out in triplicate. Percent inhibition with anti-NKG2D mAb was significantly different than values in all other groups (*p<0.001).

Vγ9Vδ2****T cells exploit different pathways for killing of tumor cells that rely on secretion of proinflammatory cytokines and proapoptotic molecules or on cell contact-dependent lysis through NK-like or TCR-dependent interactions [9]. We assessed the mechanisms responsible for killing of chemotherapy-sensitized CICs by Vγ9Vδ2****T cells, by individually blocking TCR or NKG2D receptors. Cytotoxicity of chemotherapy-pretreated colon CIC lines by two different Vγ9Vδ2****T cell lines was significantly inhibited by anti-NKG2D mAb, while the Vγ9Vδ2****TCR seems to play a minor role as indicated by the failure of anti-CD3 and anti-pan γδ TCR mAbs to inhibit cytotoxicity (**Figure 5**). In addition, Vγ9Vδ2****T cell killing of chemotherapy-sensitized targets was assessed in the presence of mevastatin, which inhibits 3-hydroxy-3-methylglutaryl-CoA and prevents zoledronate-mediated accumulation of endogenous phosphoantigens as IPP. Mevastatin failed to inhibit killing of all tested chemotherapy-pretreated colon CIC lines by two different allogeneic Vγ9Vδ2****T cell lines (**Figure 5**), thus indicating that chemotherapy-induced sensitization of CICs to Vγ9Vδ2****T cell cytotoxicity does not rely on production of mevalonate metabolites.

**Figure 6:**
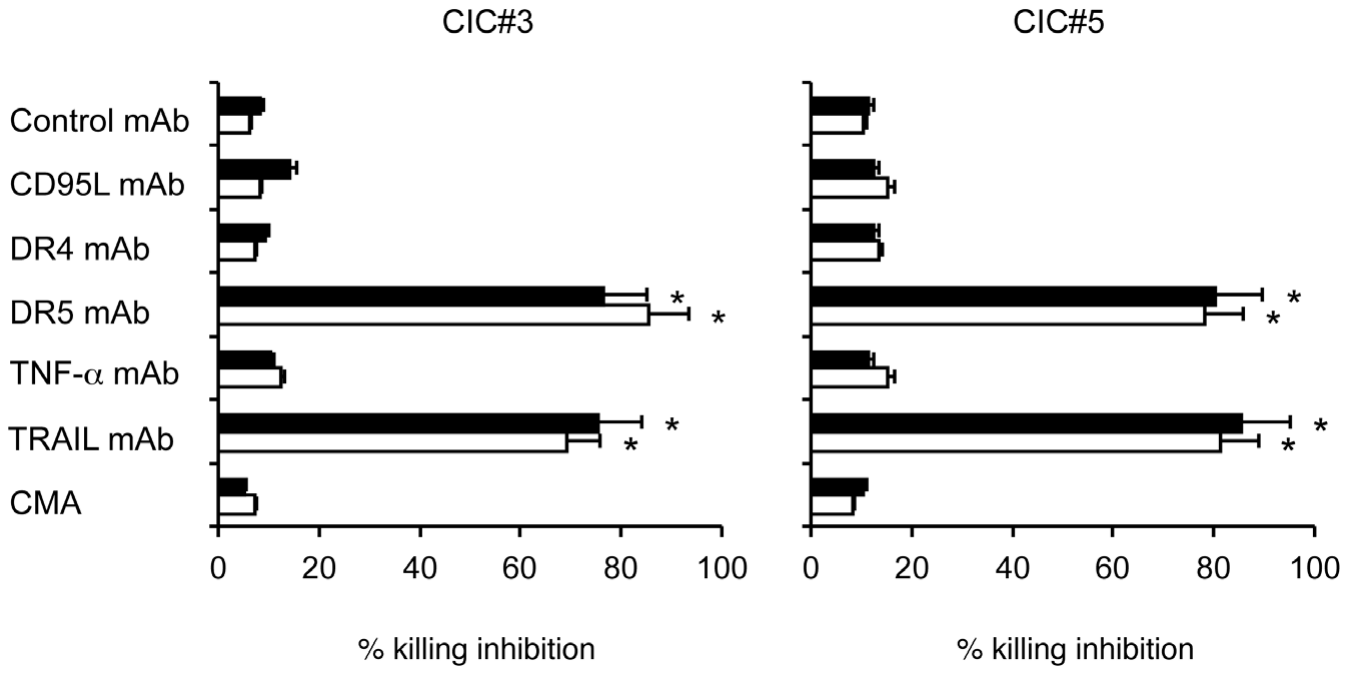
Modulation of the cytotoxic activity of Vγ9Vδ2 T cells by blocking death receptors interactions. The Vγ9Vδ2 T cell line COLD2-1 was cultured with two chemotherapy-treated colon CICs (CIC#3 and CIC#5) at an E:T ratio of 20∶1, in the presence of blocking antibodies to TNF-α, FasL (CD95L), TRAIL receptors R1 (DR4) or R2 (DR5), or concanamycin A (CMA). Specific cytotoxicity levels achieved by the Vγ9Vδ2 T cell line COLD2-1 were 61±7 for CIC#3 and 65±12 for CIC#5. Data are mean ± SD of experiments carried out in triplicate. Percent inhibition with anti-DR5 and anti-TRAIL mAbs were significantly different than values in all other groups (*p<0.001).

To further elucidate the mechanisms of killing of chemotherapy-sensitized colon CICs by Vγ9Vδ2****T cells, we individually inhibited the granule exocytosis, TNF-α-, TRAIL-, and FasL-mediated pathways. Killing-inhibition experiments revealed that Vγ9Vδ2****T cell cytotoxicity of chemotherapy-pretreated colon CIC targets was significantly inhibited by anti-DR5 mAb, whereas mAbs against DR4, TNF-α, and FasL, or treatment with CMA to block the granule-exocytosis pathway, all failed to inhibit. **Figure 6** shows representative data with two Vγ9Vδ2****T cell lines and the two colon CIC lines, CIC#2 and CIC#4.

## Discussion

It is now emerging that cancer is generated by a population of cells displaying stemness features, named cancer stem cells or cancer-initiating cells (CICs) [1], [2]. These cells, which contribute only to a minor fraction of the total tumor mass, undergo long-term expansion with retention of their ability to reproduce the original tumor phenotype, thus providing evidence for self-renewal and tumor-initiating capacity [1], [2]. The CIC population is more resistant than differentiated primary cells to conventional chemotherapy and radiotherapy and to putative innovative therapies such as those based on the use of TRAIL. This refractoriness has been attributed to the fact that CICs express multidrug resistance genes including high levels of anti-apoptotic proteins and ABC (ATP Binding Cassette) transporters which pump out the drugs, but also to the fact that chemotherapy targets dividing cells and consequently fails to kill the slow-cycling CICs [3]–[5].

Data from recent clinical studies have suggested that combining chemotherapy with immunotherapy has survival benefits than chemotherapy alone [6], [29], as outlined for example by the combination of chemotherapy and monoclonal antibodies [30]–[32]. Moreover, it is known that chemotherapeutic drugs can sensitize tumor cells to cytotoxicity mediated by CD8, NKT or Vγ9Vδ2****T cells [33] thorugh several different mechanisms [34]. However, we recently found that colon CICs are resistant to Vγ9Vδ2****T cell cytotoxicity, unless they are sensitized with zoledronate [35]: similarly, we have now tested the possibility that chemotherapeutic drugs currently used in the treatment of colon cancer might also sensitize colon CICs to Vγ9Vδ2****T cell killing.

Initial testing of cytotoxicity revealed that in analogy with our previously reported results [27], many colon CIC lines were resistant to the cytotoxic activity of Vγ9Vδ2****T cells, but pretreatment with low, sublethal concentrations of chemotherapeutic drugs 5-FU and DXR sensitizes CIC targets to Vγ9Vδ2****T cell killing, resulting in additive cytotoxicity activity.

Vγ9Vδ2****T cells interact with and kill tumor targets thorugh several different mechanisms including granule exocytosis, death receptor/ligands interactions with TNF, TRAIL and FasL, and TCR- or NKG2D-mediated recognition of phosphoantigens or stress-inducible molecules, respectively. All tested colon CIC lines constitutively expressed mRNA encoding for HLA-class I, ICAM-1, CD155, CD112, MICA/B, ULPBP1-4, Fas (CD95), TNF-R1, DR4 (TRAIL-R1) and DR5 (TRAIL-R2) molecules on their surface, but expression of all these molecules did not render CICs sensitive to Vγ9Vδ2****T cell killing. However, exposure of colon CICs to 5-FU and, although at a lesser extent DXR, significantly increased DR5 expression.

Several previously published reports in the literature have demonstrated that many chemotherapeutic drugs, including 5-FU and DXR, upregulate DR5 expression on tumor cell lines of distinct tissue origin [36]–[42]. However, this effect has been reported on differentiated cancer cells, while, to our knowledge, there is no evidence of similar DR5 upregulation on CICs. Whether or not chemotherapy-induced DR5 upregulation is restricted to colon CICs or is a general phenomenon observed on other CICs is actually under study.

Nonetheless, we found that Vγ9Vδ2****T cells exploited different mechanisms to kill CIC targets, which were strictly dependent on the way of target CICs sensitization. Regardless of whether chemotherapeutic drugs or zoledronate were used to sensitize CICs, Vγ9Vδ2****T cells killing of these targets was TCR- or NKG2D-mediated: consistent with our previous report [27] chemotherapy-sensitized colon CICs were killed following NKG2D-mediated recognition and TRAIL/DR5 interaction, while both mechanisms were dispensable to the cytotoxicity of zoledronate-sensitized colon CICs, which were almost exclusively killed by TCR-mediated interaction and the perforin/granzyme pathway.

Previous studies have highlighted the importance of NKG2D-MICA/B interactions for tumour cell recognition and effective cytotoxic activity by Vγ9Vδ2****T cells [35]–[44]. The difference between NKG2D-mediated recognition of chemotherapy-sensitized colon CICs and TCR-mediated recognition of zoledronate-sensitized CIC targets cannot be explained differential expression of MICA/B or ULBPs since neither 5-FU nor DXR changed constitutive expression levels of these molecules. It is likely that phosphoantigens production/expression by colon CICs is very low, below the threshold required for efficient recognition by the reactive Vγ9Vδ2 TCR, hence target recognition only occurs through NKG2D: the finding that colon CICs become sensitive to Vγ9Vδ2****T cell cytotoxicity upon exposure to zoledronate [27], which enhances phosphoantigen accumulation and production, supports this possibility.

We conclude that *in vivo* activation of Vγ9Vδ2****T cells or adoptive transfer of *ex vivo*-activated Vγ9Vδ2****T cells, together with or soon after administration of certain chemotherapeutic drugs may substantially increase their anti-tumor effects. Additional clinical studies are thus needed to assess the efficacy of this combinatory therapy, possibly including the novel γδ T cell-based immunotherapeutic approach that *ex-vivo* expansion of polyclonal γδ T cells followed by introduction of a CD19-specific chimeric antigen receptor render them bispecific and more efficient in killing of CD19^+^ tumor cell lines *in vitro* and in xenografts [45].

## Materials and Methods

### Peripheral Blood and Colon Cancer Samples

Human peripheral blood mononuclear cells (PBMC) and colon cancer tissues were obtained in accordance with the ethical standards of the institutional committee of human experimentation from patients undergoing a colon resection for colon adenocarcinoma. Histological diagnosis was based on microscopic features of carcinoma cells determining the histological type and grade. PBMC were isolated from colon cancer patients by density gradient centrifugation using Ficoll-Hypaque (Pharmacia Biotech, Uppsala, Sweden) and were cryopreserved in 80% RPMI 1640 (Life Technologies, Monza, Italy), 10% DMSO (Sigma, St. Louis, MO) and 10% heat-inactivated fetal calf serum (FCS, Life Technologies).

According to Italian rules (Article 13 of Legislative Decree no. 196/03), this study did not require authorisation by the local ethical committee. The study was performed in accordance to the principles of the Helsinki declaration and all individuals gave written informed consent to participate.

### Purification and Culture of CICs

Cancer tissues were extensively washed in saline buffer containing antibiotics and incubated overnight in DMEM/F12 (Life Technologies) containing penicillin (500 IU/ml), streptomycin (500 µg/ml) and amphotericin B (1.25 µg/ml) (Life Technologies). Enzymatic digestion was performed using collagenase (Life Technologies, 1.5 mg/ml) and hyaluronidase (Sigma, 20 µg/ml) in DMEM containing antibiotics/antimycotics for 1 hour. Recovered cells were then cultured in serum-free medium (DMEM/F12) supplemented with 6 mg/ml Glucose, 1 mg/ml NaHCO3, 5 mM HEPES, 2 mM L-Glutamine, 4 µg/ml Heparin, 4 mg/ml BSA, 10 ng/ml βFGF, 20 ng/ml EGF, 100 µg/ml apotrasferrin, 25 µg/ml insulin, 9,6 µg/ml putrescin, 30 nM sodium selenite anhydrous and 20 nM progesterone (Sigma) to a final concentration of 3×10^5^ cells/ml. These culture conditions select for immature tumor cells that slowly proliferate, giving rise, within 2–3 months, to tumor cell aggregates, called “spheres”. Sphere-forming cells can be propagated by enzymatic dissociation of spheres (3 mM EDTA, 50 nM DTT in PBS), followed by re-plating of single cells and residual small cell aggregates in fresh serum-free medium [28], [46], [47].

Tumorigenicity was evaluated by subcutaneous implantation of either disaggregated colon cancer sphere cells or sphere-derived differentiated cells [27]. Differentiated colon cancer cells lines DLD-1, SW620 and SW403 (American Type Culture Collection) were obtained from Dr. Ruggero De Maria (“Regina Elena” National Cancer Institute, Rome, Italy) and were maintained in DMEM containing antibiotics and 10% FCS. All cell cultures were carried out at 37°C in a 5% CO_2_ humidified incubator.

### Anti-tumor Agents, Antibodies and Reagents

The chemotherapeutic agents 5-fluorourcil (5-FU) and doxorubicin (DXR) were obtained from Sigma, through the pharmacy of the University Hospital. Drugs were diluted in DMSO and diluted to the required concentrations in PBS prior to use.

The following unconjugated, FITC-, PE-, PE-Cy5- or APC-conjugated monoclonal antibodies (mAbs) were used: anti-TCR Vδ2 (B6, BD Biosciences, San José, CA), anti-NKG2D (1D11, eBioscience, San Diego, CA), anti-CD95L (2C101,Vinci Biochem, Firenze, Italy), anti-MICA/B (6D4, BD Biosciences).

Additionally, the following purified mAbs were also used: anti-CD3 (blocking, MEM-57), anti-HLA Class I monomorphic (MEM-147) from Prof. Vaclav Horejsi (Institute of Molecular Genetics, Prague, Czech republic), anti-TCR pan γδ (IMMU510, a gift of Dr. Marc Bonneville, Institut de Biologie, Nantes, France), anti-TNF-α (Infliximab, a gift of Prof. Giovanni Triolo, Dipartimento Biomedico di Medicina Interna e Specialistica, Università di Palermo, Palermo, Italy), anti-TRAIL receptors TRAIL-R1(DR4), TRAIL-R2 (DR5), TRAIL-R3 (LIT, DcR1) and TRAIL-R4 (TRUNDD, DcR2) all provided by Dr. Henning Walczak (Tumor Immunology Unit, Division of Medicine, Imperial College, London, UK).

Concanamycin A (CMA) and mevastatin were purchased from Sigma, while zoledronate was from Novartis Pharma, Basel, Switzerland.

### Generation of Polyclonal Vγ9Vδ2 T Cell Lines

Polyclonal V**γ**9Vδ2 T cell lines were generated by first enriching PBMC using a γδ T cell isolation kit (Miltenyi Biotec, Bergisch Gladbach, Germany), followed by sorting single Vγ9Vδ2 T cells through a FACSAria (BD Biosciences) with specific mAbs. Cells (2×10^3^) were then cultured into each well of round-bottom, 96-well plates containing 2×10^4^ irradiated (40 Gy) allogeneic PBMC, 2×10^3^ irradiated (70 Gy) EBV-transformed allogeneic B cells, 0.5 µg/ml PHA (Sigma), and 200 U/ml recombinant interleukin 2 (Proleukin, Novartis Pharma). Growing lines were expanded in 200 U/ml IL-2 and restimulated every 2 weeks. Usually, cells were collected after 4–6 weeks of culture to be used for functional assays *in vitro*.

### Cytotoxic Assay

Target colon CIC (10^5^ cellsml) were pre-treated with 5-FU (2.5–250 µg/ml), DXR (0.025–2.5 µM) or zoledronate (0.5 µM) for 24, 48 or 72 hrs. Cells were extensively washed in PBS and stained with CFSE (Merck, Milano, Italy) as follows: 50 µl of CFSE were added to 1 ml of target sphere cell suspension (5×10^5^ cells/ml) in PBS to obtain the final concentration of 2.5 µM CFSE. The cells were incubated for 10 minutes at 37°C and gently mixed every 5 min. At the end of incubation, 1 ml of FBS was added to the cell suspension to stop the staining reaction and the cells were centrifuged at 600 g for 5 min at room temperature, washed twice with cold PBS and resuspended in serum-free medium.

V**γ**9Vδ2 T cell lines were resuspended at the final concentrations of 10^6^ and 2.5×10^6^ cells/ml, were added to CFSE-stained target colon CICs (1×10^5^) and co-cultures were maintained for 6 hrs a 37°C in presence of 5% of CO_2_. At the end of the incubation period, the cells were washed with PBS and stained with 20 µl of Propidium Iodide (PI, Sigma, 1 µg/ml) for 10–15 min in ice. Finally 100 µl of cold PBS were added before acquisition on a FACSCalibur cytometer (BD Biosciences). The calculation of cytolytic activity was based on the degree of reduction of viable target cells with the ability to retain CFSE and exclude PI (CFSE^high^ PI^−^), according to reference [27].

Blocking agents were used to evaluate the mechanisms of Vγ9Vδ2 T cell-mediated cytotoxicity of colon CICs. To evaluate the contribution of mevalonate metabolites tumor target cells were treated with mevastatin (25 µM for 2 h) a selective upstream inhibitor of the mevalonate pathway. After this incubation period, target cells were washed, and Vγ9Vδ2 T cells added in the presence of 25 µM mevastatin, to maintain a constant concentration of this drug during incubation because its effect is rapidly reversible [27]. To inhibit perforin-mediated cytotoxicity, Vγ9Vδ2 T cells were incubated with concanamycin A (CMA, 15 nM) for 30 min at 37°C before co-culture with target CICs, without further washing [27]. To block the relevant cytotoxic pathways, specific or isotype-control mAbs were used at 10 µg/ml final concentration just before co-incubation assay [27].

### Real-time Quantitative RT-PCR

Total RNA was extracted with the ABI PRISM 6100 Nucleic Acid PrepStation (Applied Biosystems through Life Technologies) according to the manufacturer’s instructions. Random hexamers and an MMLV Reverse Transcriptase kit (Stratagene, La Jolla, CA) were used for cDNA synthesis. Transcripts were quantified by real-time quantitative PCR on an ABI PRISM 7700 Sequence Detector (Applied Biosystems) with Applied Biosystems predesigned TaqMan Gene Expression Assays and reagents according to the manufacturer’ s instructions. The following probes were used (identified by Applied Biosystems assay identification number): HLA-A, Hs01058806_g1; HLA-B, Hs00818803_g1; HLA-C, Hs00740298_g1; ICAM-1, Mm00516023_m1; CD155, Hs00197846_m1; CD112, Hs01071562_m1; MICA, Hs00741286_m1; MICB, Hs00792952_m1; ULBP-1, Mm01180648_m1; ULBP-2, Hs00607609_mH; ULBP-3, Hs00225909_m1; Fas (CD95), Hs00236330_m1; TNF-R1, Mm00441883_g1; DR4 (TRAIL-R1), Hs00269492_m1; DR5 (TRAIL-R2), Hs00366278_m1. For each sample, mRNA abundance was normalized to the amount of 18S rRNA.

### Statistics

The two-tailed Student’s *t* test was used to compare significance of differences between groups. All values are expressed as mean ± standard deviation (SD).

## Supporting Information

Figure S1 **A low number of colon CIC spheres retain the capacity to form a tumor when injected s.c. into immunodeficient mice.** Subcutaneous tumor growth in NOD/SCID mice 10 weeks after injection of 2000 disaggregated cells from colon cancer spheres. One representative experiment of two performed with cells from different donors is shown.
(TIF)
