# Supplementary figures and images for: Chemotherapy Sensitizes Colon Cancer Initiating Cells to Vγ9Vδ2 T Cell-Mediated Cytotoxicity
Source: PLoS One. 2013 Jun 6;8(6):e65145. doi: 10.1371/journal.pone.0065145 (PMC3675136; doi:10.1371/journal.pone.0065145)

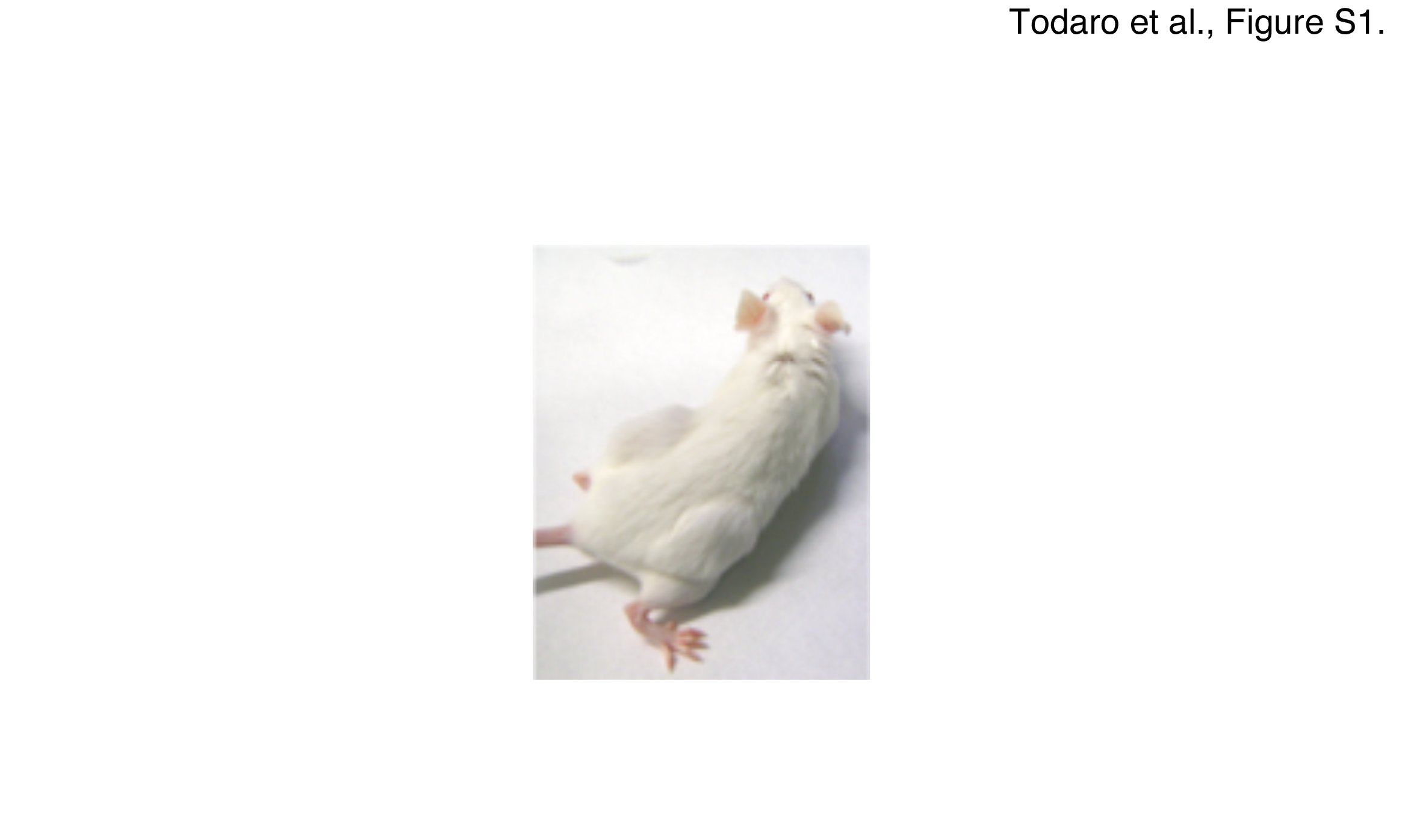

Supplement: Figure S1 — A low number of colon CIC spheres retain the capacity to form a tumor when injected s.c. into immunodeficient mice. Subcutaneous tumor growth in NOD/SCID mice 10 weeks after injection of 2000 disaggregated cells from colon cancer spheres. One representative experiment of two performed with cells from different donors is shown. (TIF) [file pone.0065145.s001.tif]
